# Transient inconsistency between population density and fisheries yields without bycatch species extinction

**DOI:** 10.1002/ece3.6868

**Published:** 2020-10-10

**Authors:** Renfei Chen

**Affiliations:** ^1^ School of Life Science Shanxi Normal University Linfen China

**Keywords:** bycatch, marine reserves, population persistence, transient dynamics

## Abstract

Recent studies have demonstrated the great advantages of marine reserves in solving bycatch problems by maintaining the persistence (i.e., avoid extinction) of endangered species without sacrificing the fisheries yields of target species. However, transient phenomena rather than equilibrium states of population dynamics still require further research. Here, with a simple and general model, the transient dynamics of the target fish species are investigated under management which minimizes extinction risk of the bycatch species. An interesting finding is that fisheries yields can strongly fluctuate even if population density both inside and outside marine reserve only slightly varies (or vice versa), leading to transient inconsistency between the population densities and fisheries yields. This finding suggests that population density dynamics of the target fish species cannot be used to predict the transient phenomena of fisheries yields (or vice versa) in fisheries management. However, the unpredictability can be receded as the sensitivity analyses show that a large marine reserve size and low escapement rate can shorten the transient duration.

## Graphical abstract

Transient phenomena rather than equilibrium states of population dynamics still require further research for marine reserve management. With a simple and general model of transient dynamics of strong stock fish species, an interesting finding is that fisheries yields can fluctuate even if population density both inside and outside marine reserve only slightly varies, leading to transient inconsistency between the population densities and fisheries yields.

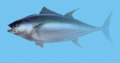

## 1 INTRODUCTION

Marine reserves have been used as an effective tool to protect the endangered species and achieve sustainable fisheries yields and other benefits (Edgar et al., 2014; Goetze et al., 2018; Herrera et al., 2016; White et al., 2008). To improve the functions of marine reserves, many marine ecologists have devoted tremendous effort to the design of marine reserves (Gerber et al., 2003; Guichard et al., 2004; Mangel, 2000; Sanchirico et al., 2006). One of the greatest debates in marine reserve design is whether permanent marine reserves can improve fisheries yields while maintaining the persistence of the target population (Game et al., 2009; Hastings & Botsford, 1999; Hastings et al., 2017; Hilborn, 2017; Kaplan et al., 2010). A simple theoretical model indicated that, without sacrificing the persistence of the target species, the implementation of a permanent marine reserve with reasonable assumptions could achieve fisheries yields equivalent to those obtained with fisheries management methods of harvesting a fixed amount of fish resources without marine reserves (Hastings & Botsford, 1999). However, the model is suitable for only one species without considering the nontarget species.

The incidental capture of nontarget species, which is called “bycatch,” is a great threat to fisheries sustainability, especially for some endangered marine species (Komoroske & Lewison, 2015; Scales et al., 2018; Taylor et al., 2017; Welch et al., 2018). Therefore, one main challenge in fisheries management is simultaneously achieving two important goals: (a) improving the harvested yield of target fish species so that to benefit fishers; and (b) avoiding the extinction of the endangered species (which is usually captured as bycatch) so that to maintain biodiversity and achieve ecological conservation aims. A recent theoretical framework suggested that marine reserves could improve the fisheries yields of the strong stock (i.e., the target species. In most cases, they have high fecundity and can be easily persistent even under the stress of fishing) while maintaining the persistence of the weak stock (the bycatch species which has low fecundity and more easily goes extinct), even when both strong and weak stocks are captured at the same rate (Hastings et al., 2017). However, the transient dynamics (which study the dynamics on ecological time scales that may be different from asymptotic dynamics) of the two‐species system are still not clear, which may become a major barrier to applying the theoretical framework of marine reserve design to empirical conservation management.

The application and importance of transient dynamics are emphasized by the shortcomings of long‐term asymptotic population dynamics. Transient analyses pay attention to dynamical systems over a short timescale rather than systems in the equilibrium state after a sufficiently long time (Feng et al., 2019; Mari et al., 2019; Rudolf, 2019; Shriver et al., 2019). If the dynamics of a certain system depend on the timescale of the analysis, then the transient dynamics may be much different from the dynamics when the system achieves stability. For example, in a two‐species system (one native species and an invasive species), the two species may coexist for a long time (because of the long transient times) even though the invasive species will exclude the native species in the asymptotic equilibrium state (Hastings et al., 2018). Thus, empirical data of such a system monitored on an intermediate timescale will lead to incorrect judgments and management in species conservation. Conversely, if a population has a very low density at the intermediate timescale but very high density at the asymptotic equilibrium state, analysis from only asymptotic dynamics will lead to the conclusion (which can be avoided if we study the transient dynamics of a system) that the population can persist, missing the fact that the population faces a high risk of extinction on ecological short timescales (Hastings, 2004; Hopf et al., 2016a; Nickols et al., 2019). The underlying illustration of the difference between transient and asymptotic dynamics is that the ecological system can exhibit abrupt change even without changing environmental conditions (i.e., parameters in a model). Without investigating transient dynamics, a similar case in fisheries would lead to overfishing because it is misunderstood that the population can persist even at low density.

Recent reviews indicate that there are five general scenarios in which transient phenomena may arise. Among which three of them are ghost, crawl‐by, and slow–fast systems (Hastings et al., 2018; Morozov et al., 2019). If a system is stable within a tipping point and becomes unstable beyond the tipping point (which is a bifurcation phenomenon beyond a tipping point of a system), the dynamics of the system (even if it does not possess an equilibrium point in the long term) will mimic a system's dynamics that have an equilibrium state (i.e., attractor). Such a case is called “ghost” or “ghost attractor” (Hastings et al., 2018). The population dynamics of a system with a ghost may spend a long time around the ghost, and thus long transient phenomena may occur. If a system spends a long time in the vicinity of the unstable equilibrium point caused by a saddle‐type invariant set, then the transient is called “crawl‐by.” The definitions of the transient dynamics of a system caused by “crawl‐by” are very similar to those caused by a “ghost.” The difference between a ghost and crawl‐by is that a system with ghosts may or may not have attracting directions because of the lack of invariant sets, and the initial state of a ghost system should be around the ghost while a crawl‐by system should have saddles (that is unstable equilibria for a system) and thus always have attracting directions (Hastings et al., 2018; Morozov et al., 2019). A fast–slow system is characterized by different multiple timescales in the system (Bertram & Rubin, 2017). For example, in a resource–consumer system, insects that feed on trees have a much shorter lifetime than the trees. Thus, the population dynamics of trees change very slowly (assumed to be fixed in ideal systems), even when insects have gone through several dynamical generations (Hastings et al., 2018; Rinaldi & Scheffer, 2000).

Supposing a system describing the fished population densities both inside and outside marine reserves, the existence of ghost transients will lead to the population staying a long time at certain unstable density level if the initial states approach that density level, and the existence of crawl‐by transients will lead to the oscillations of population densities by making the system approaches the unstable equilibrium (specifically, the saddle point) first and then leaves. Transients caused by a fast–slow system can probably be observed in a two‐species fisheries management system in which the target fast‐growing species is the fast component and the slow‐growing endangered bycatch species is the slow component. If these types of transients exist in a fisheries system with reserve implementation, detecting the dynamics of population density and fisheries yields will be difficult as suggested by the transient analyses on fisheries before and after reserve establishment (Kaplan et al., 2019; Nickols et al., 2019), and a clear understanding of their transients is necessary to increase the detectability in fisheries management. In this paper, all three types of transients mentioned above are investigated to show transient dynamics in fisheries yields and population density.

Although transient phenomena have been demonstrated and classified by increasing evidence both empirically and theoretically (Hastings, 2001, 2004; Hastings & Higgins, 1994; Morozov et al., 2019) and its applications in marine reserve management and policy‐making is increasing (Hopf et al., 2016a, 2016b; Kaplan et al., 2019; White et al., 2013), the transient research in marine reserve design is still limited. The research in this paper specifically focuses on the transient dynamics of the discrete model (the definitions of all the symbols in the models can be seen in Table 1) developed by Hastings et al. (2017) to describe a two‐species system consisting of an endangered bycatch species with low fecundity and a target fish species with high fecundity. The intrinsic mechanisms in the model that leads to the transient analysis here derive from the fact that, in the model (Hastings et al., 2017), the adult survivals, as well as the interplay (i.e. ecological connection) between population density inside and outside the marine reserve, lead to complex trajectories in the variation of both fisheries yields and population densities (which will be explained further in the paper). This theoretical framework is based on several main assumptions. First, this framework assumes that adult fish are relatively stationary while larvae are mobile and widely distributed. Second, it assumes that different species considered in the system are subject to the same fisheries management practices with the same capture rates outside marine reserves and the same protection inside marine reserves. Third, no further complex factors, such as time delay and age structure, are considered in the system. Fourth, there is no ecological connection between the bycatch and the fished species. With these assumptions, I study the transient dynamics that may result from ghost attractor, crawl‐by, and the fast–slow systems with different timescales. Both analytical and numerical approaches are used to investigate the transient phenomena that may be concealed in the target system. With an analytical approach, whether saddle points (describing population density both inside and outside marine reserves) exist is discussed so that to insight into which types (ghost attractor or crawl‐by) cause the transient dynamics. Further, I also analytically discuss the assumption that the target system is a fast–slow system (i.e., multiple time scales existing in a system) to determine whether there is Hopf bifurcation for the fast components of the system. Last, with the numerical method, simulations are performed to study the transient dynamics of both population density and fisheries yields with both the initial population density at the saddle points and a random initial population density. In addition, sensitivity analysis is used to investigate how fisheries management (the design of marine reserve size and the escapement rate) affect the detectability in population dynamics. This paper aims to answer two questions: (a) How fisheries yields and population densities of targeted species vary with transient time scales under the persistence of bycatch species? (b) Whether and when population density can be used to predict fisheries yields?

**Table 1:** Definitions of the symbols used in this paper

Symbols	Description
*a*	The survivorship of adults of the strong stock species (*a*_w_ for the weak stock species).
*m*	Per capita fecundity for the strong stock species (*m*_w_ for the weak stock species).
*α*	Proliferation rate per generation in the Beverton–Holt growth function for the strong stock species (*α*_w_ for the weak stock species).
*β*	Carrying capacity in the Beverton–Holt growth function for the strong stock species (*β*_w_ for the weak stock species).
*c*	The fraction of the coastline in a no‐take marine reserve for both the strong and weak stock species.
*E*	The escapement rate representing the fraction of the fish stock that is left unharvested outside marine reserves for both the strong and weak stock species.
$n_{t}^{i}$	Population density inside (*i* = R) and outside (*i* = O) marine reserves at time *t* for the strong stock species.
*Y*_P_	The harvested yield of the strong stock species.
***A***	Population projection matrix of the Equations 1 and 2.
***N_t_***	2 × 1 vector of population density at time *t*.
***N***_0_	Initial conditions of the densities both inside and outside marine reserves.
*w*_1_	The dominant right eigenvector of matrix ***A***.
*θ*	The transient metric representing the similarity between the initial conditions and stable equilibrium state.
*ρ*	The transient metric representing the rate of convergence to the asymptotic equilibrium state.
*λ*_1_, *λ*_2_	Two eigenvalues of the Jacobian matrix of Equations 1 and 2.
*λ*_3_, *λ*_4_	The first and second eigenvalues of ***A***, respectively.

## 2 MODEL AND ANALYSIS

The model analyzed here (whose management goal is to maximize fisheries yields rather than economic welfare) was derived from recent research on bycatch problems (Hastings et al., 2017). I focus on two species, one called the strong stock (the target species in the fishery) and the other called the weak stock (an endangered species that could easily become extinct). To simplify the problem, I use similar approaches to those used in previous research (Hastings et al., 2017) and only study the population dynamics of the strong stock under the conditions in which the weak stock is persistent rather than studying the population dynamics of both species simultaneously (because, in most cases, the strong stock is the primary target in a fishery and is expected to achieve maximum yields while the weak stock species is the secondary target in the fishery and will much easier become extinct). The population dynamics of the strong stock are described by keeping track of the densities inside and outside of marine reserves. Based on the assumptions that are the same as those in Hastings et al. (2017), the density dynamics for the strong stock is described as follows:

(1) $$n_{t+1}^{R}=n_{t}^{R}a+f\left(m\left(cn_{t}^{R}+\left(1‐c\right)n_{t}^{O}\right)\right)$$

(2) $$n_{t+1}^{O}=\left[n_{t}^{O}a+f\left(m\left(cn_{t}^{R}+\left(1‐c\right)n_{t}^{O}\right)\right)\right]E$$

where $n_{t}^{R}$ and $n_{t}^{O}$ represent the density of the strong stock inside and outside marine reserves at time *t*, respectively. The function *f*(·) shows the survival of young fish individuals until they recruit to the adult population. The parameters *m*, *a*, *c,* and *E* describe the per capita fecundity, the survivorship of adults, the fraction of the coastline in a no‐take marine reserve, and the escapement rate representing the fraction of the fish stock that is left unharvested, respectively. All the variables and parameters exhibited here are for the strong stock, whose specific values are different from those for the weak stock. However, for both species in the same system, the marine reserve size (i.e., the length of marine reserve coastline because the research here is one dimensional) and the escapement rate are the same. Thus, *c* and *E* are also used for the weak stock. Accordingly, the harvested yield of the strong stock produced from such a system (Equations 1 and 2) is:

(3) $$Y_{t+1}=\left[n_{t}^{O}a+f\left(m\left(cn_{t}^{R}+\left(1‐c\right)n_{t}^{O}\right)\right)\right]\left(1‐c\right)\left(1‐E\right)$$

As for the weak stock, I use the same symbols as those for the strong stock but add a subscript “w” for distinction (see Table 1 for the definitions of all the symbols). To achieve the weak stock persistence condition, it is assumed that the dynamics of the weak stock is unstable when population density is zero. Let the determinant of the following Jacobian matrix *J* be zero:

(4) $$J=\left[\begin{array}{cc} f_{w}^{\prime }\left(0\right)m_{w}c+a_{w}‐1 & f_{w}^{\prime }\left(0\right)m_{w}\left(1‐c\right)\\ Ef_{w}^{\prime }\left(0\right)m_{w}c & E\left[a_{w}+f_{w}^{\prime }\left(0\right)m_{w}\left(1‐c\right)\right]‐1 \end{array}\right]$$

which derives from the fact that the persistence boundary for the weak stock is achieved by the condition that the determinant of the matrix *J* is zero as suggested by previous research (Hastings et al., 2017). Note that Equation 4 is a steady‐state condition. Then, we have the weak stock persistence condition:

(5) $$E=\frac{a_{w}‐1+\alpha_{w}m_{w}c}{\left(a_{w}‐1\right)\left(a_{w}+\alpha_{w}m_{w}\right)+\alpha_{w}m_{w}c}$$

The calculation of Equation 5 is based on the Beverton–Holt functional form for *f*(·):

(6) $$f\left(n\right)=\frac{\alpha_{w}n}{1+\frac{n}{\beta_{w}}}=\frac{\alpha_{w}\beta_{w}n}{\beta_{w}+n}$$

where *α*_w_ and *β*_w_ denote the proliferation rate per generation and carrying capacity, respectively, and thus $f_{w}^{\prime }\left(0\right)=\alpha_{w}$ (other similar information about Equations (1), (2), (3), (4), (5), (6) can be seen in Hastings et al. (2017)). Note that Equation 5 is a necessary but not sufficient persistence condition as the weak stock may have the too low density to persist by considering the stochasticity in the real‐world system.

**Figure 1:**
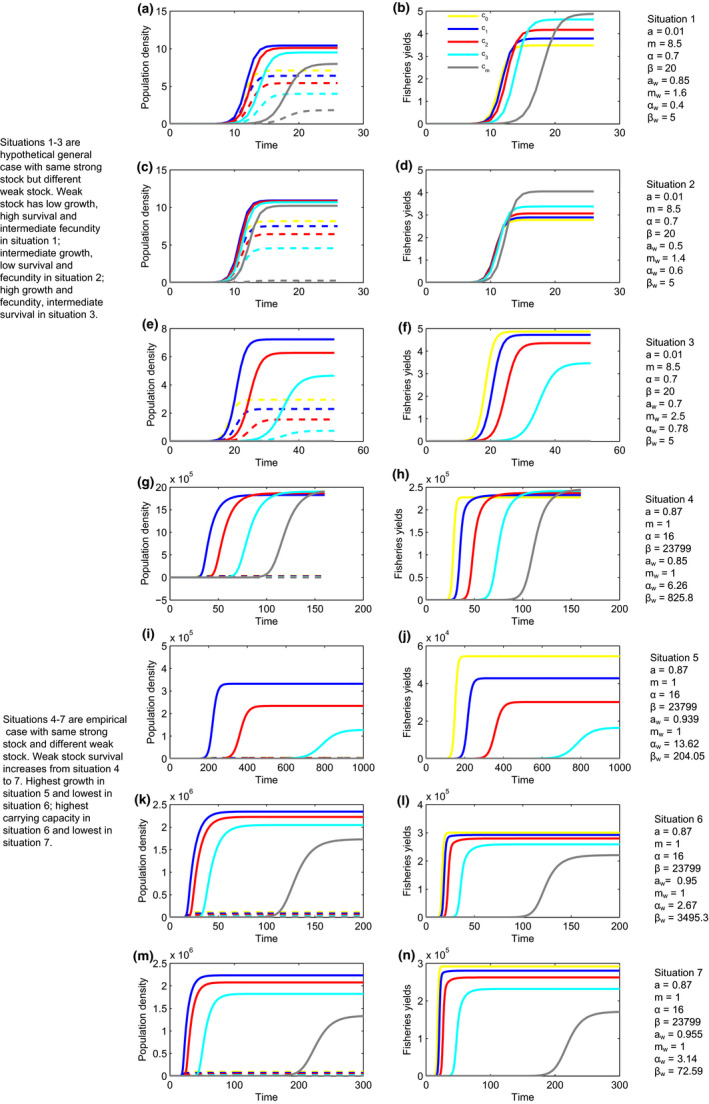
The population density dynamics and fisheries yields with the initial density both inside and outside the reserves at very low level for the target fish species. Different colors represent different gradients of the length of marine reserve coastline (i.e., marine reserve size) marked as *c*_0_, *c*_1_, *c*_2_, *c*_3_, and *c_m_* (*c*_0 _= 0; *c*_1_ < *c*_2_ < *c*_3_; *c_m_* approaches the maximum value. The legend is shown in the subplot (b), which is also suitable for other subplots). Note that the specific values of *c*_1_, *c*_2_, *c*_3_, and *c_m_* are different in the different subplots, although *c*_0 _= 0 remains the same. *E* is calculated based on Equation 5. The solid and dashed lines in the subplots of population density denote the density inside and outside marine reserves, respectively. (a, b) Simulation results for situation 1 with *c*_1_ = 0.050, *c*_2_ = 0.100, *c*_3_ = 0.150, and *c_m_* = 0.200 (specific values for the other parameters can be seen in Table 2). (c, d) Simulation results for situation 2 with *c*_1_ = 0.148, *c*_2_ = 0.295, *c*_3_ = 0.443, and *c_m_* = 0.590. (e, f) Simulation results for situation 3 with *c*_1_ = 0.035, *c*_2_ = 0.070, *c*_3_ = 0.105, and *c_m_* = 0.140. (g, h) Simulation results for situation 4 with *c*_1_ = 0.006, *c*_2_ = 0.012, *c*_3_ = 0.018, and *c_m_* = 0.024. (i, j) Simulation results for situation 5 with *c*_1_ = 0.0003, *c*_2_ = 0.0006, *c*_3_ = 0.0009, and *c_m_* = 0.0012. (k, l) Simulation results for situation 6 with *c*_1_ = 0.0045, *c*_2_ = 0.0090, *c*_3_ = 0.0135, and *c_m_* = 0.0180. (m, n) Simulation results for situation 7 with *c*_1_ = 0.0035, *c*_2_ = 0.0070, *c*_3_ = 0.0105, and *c_m_* = 0.0140

By analytically solving the equilibrium state for the strong stock, three solutions (i.e., three scenarios in biological meaning) can be achieved (see A.6–A.11 in Appendix S1 for details of all analyses). In scenario 1 (i.e., $n_{t}^{R}=0$, $n_{t}^{O}=0$), the equilibrium of the system will stay at very low density level both inside and outside marine reserves. In scenario 2 (i.e., $n_{t}^{R}=\frac{mc\alpha \beta +\left(a‐1\right)\beta }{\left(1‐a\right)mc}$, $n_{t}^{O}=0$), fishing effort outside the reserves is extremely strong, such that the escapement rate approaches zero (i.e., “scorched earth” assumption). In such a case, the sustainable fisheries yields all depend on the larvae dispersal from marine reserves to the harvested area, and population density inside the marine reserves could be high and the marine reserve size should be large enough to maintain species persistence. In scenario 3 (i.e., $n_{t}^{R}=\frac{\alpha \beta }{1‐a}‐\frac{\beta \left(1‐Ea\right)}{mc\left(1‐Ea\right)+m\left(1‐c\right)\left(1‐a\right)E}$, $n_{t}^{O}=\frac{\alpha \beta E}{1‐Ea}‐\frac{\beta E\left(1‐a\right)}{mc+m\left(1‐a‐c\right)E}$), the system approaches equilibrium with density both inside and outside the marine reserves maintaining at a certain high level, which turns out to be stable. Further analyses indicate no Hopf bifurcations (which is one of the approaches that leads to transient dynamics) occur with the three solutions if the system consists of Equations 1 and 2 is regarded as the fast components of the fast–slow systems (see the explanation in the section of ‘Transient analysis by considering fast‐slow systems’ in Appendix S1). However, among the three equilibrium solutions, the point ($n_{t}^{R}=0$, $n_{t}^{O}=0$) where densities are very low both inside and outside marine reserves turns out to be a saddle point (the detail can be seen in the section of “Transient analysis by finding a saddle point” in Appendix S1), which suggests that the population density for the strong stock inside and outside the marine reserve will finally leave the very low level and approach equilibriums with high densities even if population densities initially are very low. Meanwhile, although the population density varies fast when they are far away from the saddle where densities both inside and outside the reserves are very low, the system of Equations 1 and 2 will stay a long time at very low density level both inside and outside marine reserves (Figure 1), which will lead to transient dynamics. In summary, by putting the mathematical analysis in a fisheries‐relevant context, the analyses show that although there is a high equilibrium of population density both inside and outside the reserve in the end, it is reached only after a long period of low density if initial population density is too low. The analysis results show important ecological implications: the population densities inside and outside the marine reserve cannot be too low simultaneously (i.e., both of them approach zero and thus stay in the vicinity of the point ($n_{t}^{R}=0$, $n_{t}^{O}=0$) to achieve the goal of species persistence and high harvested yields. Otherwise, the fishers must be subject to low harvested yields for a long time even if the species does not go extinct (in fact, it faces high risk of extinction in this case). Although it is known that low density erodes species persistence, the analyses here provide explanations from transient perspectives.

## 3 TWO METRICS FOR TRANSIENT ANALYSIS

My analytical analysis results indicate that transient phenomena may occur when the initial densities both inside and outside marine reserves are very low (i.e., scenario 1) rather than the other two situations in which either initial densities inside or outside the reserves or both are at high level (i.e., scenarios 2 and 3). Thus, I further study two transient metrics only in scenario 1 (i.e., the initial density both inside and outside the marine reserve is very low). Using fishery‐relevant terms, this analysis will provide insight into the transient dynamics when the fishery is recovering from a highly depleted state (i.e., near‐zero population density). By considering the linear form of the density dynamics of the strong stock species, let *f*(*n*) = *n* (note that $n=m\left(cn_{t}^{R}+\left(1‐c\right)n_{t}^{O}\right)$ in Equations 1 and 2). Thus, Equations 1 and 2 turn out to be:

(7) $$N_{t+1}={\mathrm{AN}}_{t}$$

where the symbols are defined as the following:

(8) $$N_{t}=\left[\begin{array}{c} n_{t}^{R}\\ n_{t}^{O} \end{array}\right]$$

(9) $$N_{t+1}=\left[\begin{array}{c} n_{t+1}^{R}\\ n_{t+1}^{O} \end{array}\right]$$

(10) $$A=\left[\begin{array}{cc} mc+a & m\left(1‐c\right)\\ \mathrm{mcE} & \left[m\left(1‐c\right)+a\right]E \end{array}\right]$$

The first transient metric is used to calculate the similarity between the initial conditions and a stable equilibrium state. It measures how close the trajectories (initial) of population density and fisheries yields to the stable equilibrium level. According to previous research (Kaplan et al., 2019; White et al., 2013), this metric can be expressed as an angle θ between vectors ***N*_0_** and ***w*_1_**:

(11) $$\theta =\arccos\left(\frac{N_{0}\cdot w_{1}}{\left|\right|N_{0}\left|||\right|w_{1}\left|\right|}\right)$$

where ***N*_0_** represents the initial density, ***w*_1_** is the dominant right eigenvector of matrix ***A***, and the double vertical bars denote the vector norm. Smaller θ suggests that the initial trajectories of population density and fisheries yields are closer to the stable level so that to exhibit lower‐amplitude oscillations of population dynamics.

The second transient metric is used to show the rate of convergence to an asymptotic equilibrium state. Similar to previous research (Kaplan et al., 2019; White et al., 2013), this metric is expressed as *ρ* by approximately calculating the ratio of the first and second eigenvalues of matrix ***A***:

(12) $$\rho \approx \frac{\lambda_{3}}{\left|\lambda_{4}\right|}$$

where *λ*_3_ and *λ*_4_ are the first and second eigenvalues of ***A***, respectively. Smaller values of *ρ* indicate the transient behavior of population density and fisheries yields lasts longer because of the slow rate of approaching stable level. With these equations, simulations are performed, and the numerical analysis results are presented in the next section.

## 4 POPULATION TRANSIENT DYNAMICS WITH RANDOM INITIAL DENSITY

The random initial density should also be considered rather than only considering the initial density at the equilibria to investigate the transient phenomenon in the strong stock population dynamics. First, 500 (enough to show the variance of the simulation results) random numbers are produced for both population densities inside and outside the marine reserve, respectively. The range of the random initial densities is from zero to the carrying capacity. Second, the transient metric *θ* is calculated based on the random initial densities. Finally, the random initial densities that correspond to the maximum value of *θ* are chosen for further transient dynamic analysis so that the transient phenomena can be observed more easily. This derives from the fact that large value of *θ* corresponds to strong perturbations of a system as suggested by previous research (White et al., 2013), and thus, the maximum value of *θ* gives the furthest from the stable conditions.

## 5 NUMERICAL APPROACH

The numerical simulation is performed based on the assumption that the dynamics are discrete so that iteration in the computer is the best way to solve the problem. To perform the iteration, specific parameter values are needed first. The parameter values for the life‐history traits (see Table 2) are cited from previous research on the system defined by Equations 1 and 2 (Hastings et al., 2017). Seven situations are separated based on the hypothetical parameter values (situations 1–3) and parameters estimated from the West Coast groundfish fisheries (situations 4–7). The differences among situations 1–3 and the differences among situations 4–7 result from life‐history traits for the weak stock (e.g., the per capita fecundity for the weak stock is different among situations 1–3, while the adult survival for the weak stock is different among situations 4–7). In other words, different situations are set to explore the transient dynamics of the same strong stock species under the persistence of different weak stock species for both hypothetical general case and empirical case. Specifically, in hypothetical general case with a same strong stock species, the weak stock species has low proliferation rate, high adult survival, and intermediate fecundity in situation 1; intermediate proliferation rate, low adult survival, and low fecundity in situation 2; and high proliferation rate, intermediate adult survival, and high fecundity in situation 3. The same strong stock species in the empirical case is Dover sole (*Microstomus pacificus*), while the weak stock species are bocaccio (situation 4), darkblotched rockfish (situation 5), Pacific Ocean perch (situation 6), and yelloweye rockfish (situation 7), respectively. The adult survival of the weak stock increases from situation 4 to situation 7. Meanwhile, among situations 4–7, the proliferation rate of the weak stock is highest in situation 5 and lowest in situation 6, while the carrying capacity is highest in situation 6 and lowest in situation 7. By doing so, the analysis can show the transient dynamics of the strong stocks from the Eastern Pacific groundfish fishery in the United States, which is highly diverse and has historically been plagued by reserves because of overfished weak stocks. Note that bocaccio in situation 4 may not be a true weak stock as suggested by previous research (Hastings et al., 2017), which may lead to some different dynamics in comparison with the other situations. According to the specific parameter values for the life‐history traits, the value range for the marine reserve size (*c*) can be calculated with the general constraint conditions (see details in the section of “The value ranges for the parameters” in Appendix S1). For each situation, five specific values of *c* are obtained in the value range including the smallest value *c*_0_ = 0 and the biggest value *c_m_* (which approaches the maximum value of *c* in the value range) so that the simulation result is representative. Further, with specific parameter values for the life‐history traits and the value of *c*, the escapement rate (*E*) can be calculated by the weak persistence condition in Equation 5. For the scenario that initial population densities are zero both inside and outside the marine reserve (i.e., $n_{t}^{R}=0$, $n_{t}^{O}=0$), the numerical calculation through iteration denotes the dynamics of a population that recovering from heavy depletion such as the case that fish individuals in overfished harvested area are protected with the establishment of new marine reserve (mathematically, the heavy depletion corresponds to a point that stays in the very vicinity of the point ($n_{t}^{R}=0$, $n_{t}^{O}=0$) but not the point ($n_{t}^{R}=0$, $n_{t}^{O}=0$)). Specifically, both density inside and outside the reserve is set as 0.000001 rather than zero.

**Table 2:** Specific parameter values used for the simulation analyses

Parameters	Situation 1	Situation 2	Situation 3	Situation 4	Situation 5	Situation 6	Situation 7
*a*	0.01	0.01	0.01	0.87	0.87	0.87	0.87
*m*	8.5	8.5	8.5	1	1	1	1
*α*	0.7	0.7	0.7	16	16	16	16
*β*	20	20	20	23,799	23,799	23,799	23,799
*a*_w_	0.85	0.5	0.7	0.85	0.939	0.95	0.955
*m*_w_	1.6	1.4	2.5	1	1	1	1
*α*_w_	0.4	0.6	0.78	6.26	13.62	2.67	3.14
*β*_w_	5	5	5	825.8	204.05	3,495.3	72.59

Note

By applying the values to different species (including both strong and weak stocks species) in a natural system, the parameter values are classified into seven groups marked as situations 1–7. Situations 1–3 denote the hypothetical parameter values, and situations 4–7 denote parameters estimated from the West Coast ground fish fisheries. All the parameter values are the same as those in previous research that studied the asymptotic behavior of the target system (Hastings et al., 2017).

## 6 NUMERICAL RESULTS

**Figure 2:**
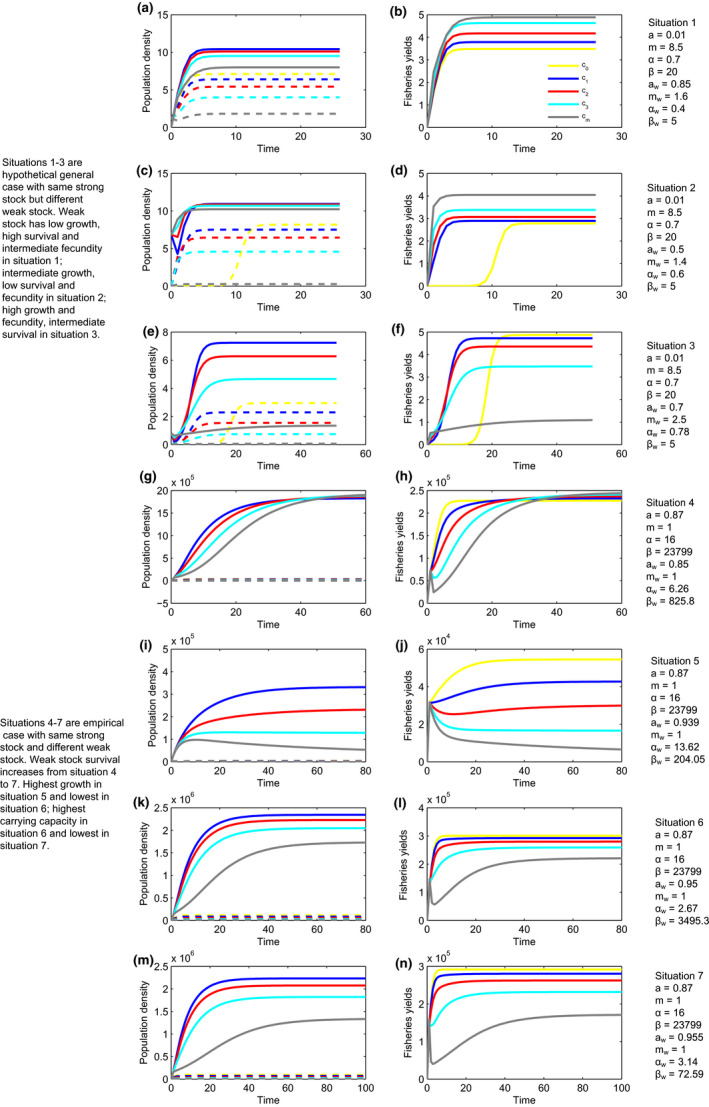
The transient phenomena of population density and fisheries yields with random initial density for the target fish species. Different colors represent different gradients of the length of marine reserve coastline (i.e., marine reserve size) marked as *c*_0_, *c*_1_, *c*_2_, *c*_3_, and *c_m_* whose specific meanings are the same as those explained in Figure 1. Note that the specific values of *c*_1_, *c*_2_, *c*_3_, and *c_m_* are different in the different subplots, although *c*_0_ = 0 remains the same. *E* is calculated based on Equation 5. The solid and dashed lines in the subplots of population density denote the density inside and outside marine reserves, respectively. The random initial density values used here are ones that gave the largest *θ*. (a, b) Simulation results for situation 1 with *c*_1_ = 0.050, *c*_2_ = 0.100, *c*_3_ = 0.150, and *c_m_* = 0.200 (specific values for other parameters can be seen in Table 2). (c, d) Simulation results for situation 2 with *c*_1_ = 0.148, *c*_2_ = 0.295, *c*_3_ = 0.443, and *c_m_* = 0.590. (e, f) Simulation results for situation 3 with *c*_1_ = 0.035, *c*_2_ = 0.070, *c*_3_ = 0.105, and *c_m_* = 0.140. (g, h) Simulation results for situation 4 with *c*_1_ = 0.006, *c*_2_ = 0.012, *c*_3_ = 0.018, and *c_m_* = 0.024. (i, j) simulation results for situation 5 with *c*_1_ = 0.0003, *c*_2_ = 0.0006, *c*_3_ = 0.0009, and *c_m_* = 0.0012. (k, l) simulation results for situation 6 with *c*_1_ = 0.0045, *c*_2_ = 0.0090, *c*_3_ = 0.0135, and *c_m_* = 0.0180. (m, n) Simulation results for situation 7 with *c*_1_ = 0.0035, *c*_2_ = 0.0070, *c*_3_ = 0.0105, and *c_m_* = 0.0140

**Figure 3:**
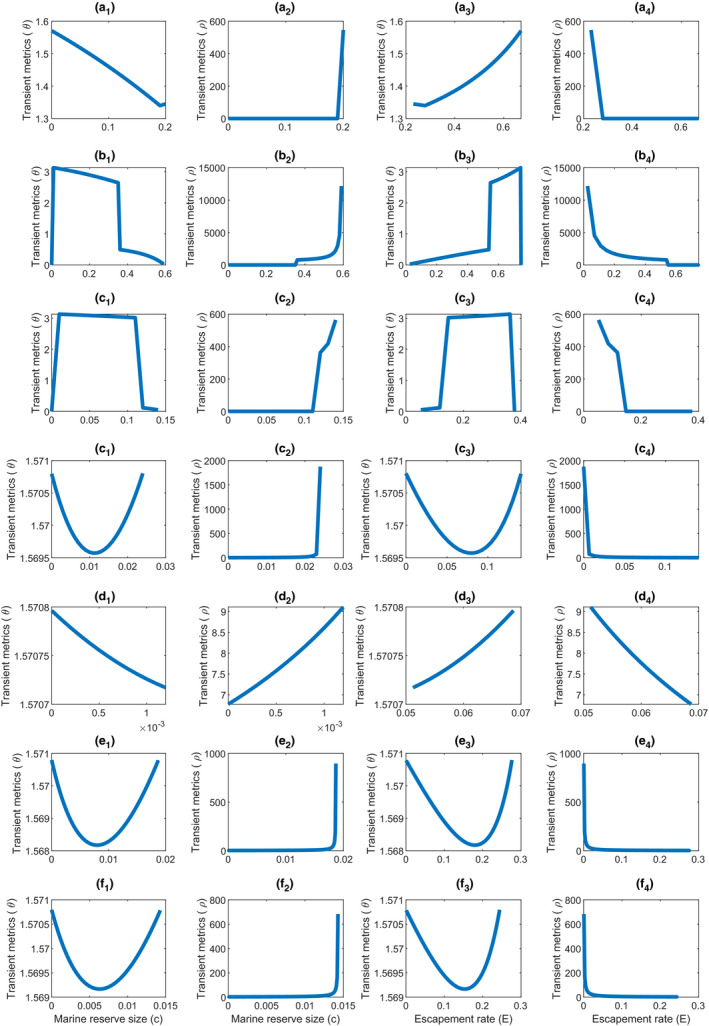
Sensitivity of two transient metrics to variations in fisheries management with a random initial density for situations 1–7 for the target fish species. a_1_, b_1_, c_1_, d_1_, e_1_, f_1_, and g_1_, variations of transient metric *θ* in response to marine reserve size; a_2_, b_2_, c_2_, d_2_, e_2_, f_2_, and g_2_, variations of transient metric *ρ* in response to marine reserve size; a_3_, b_3_, c_3_, d_3_, e_3_, f_3_, and g_3_, variations of transient metric *θ* in response to escapement rate; and a_4_, b_4_, c_4_, d_4_, e_4_, f_4_, and g_4_, variations of transient metric *ρ* in response to escapement rate

The numerical results of the fishery targeted species have shown how fisheries yields and population densities vary in transient timescales (Figures 1 and 2) and how the transient dynamics are affected by marine reserve size and the escapement rate (Figure 3), which can be used to judge when population densities can be used to predict fisheries yields. For the dynamics starting from low density level, the population densities and fisheries yields stay at a low level at the beginning of the trajectories for a long time both inside and outside the marine reserve (Figure 1), which may impair population persistence as influencing factors in real word such as Allee effects can lead to a high risk of extinction. Moreover, the great difference between initial low population density and eventually high equilibrium suggests the importance of insight into transient dynamics rather than only considering the asymptotic population density. Based on sensitivity analysis, the phenomenon that staying a long time with very low density both inside and outside the reserves (i.e., in the vicinity of the point ($n_{t}^{R}=0$, $n_{t}^{O}=0$)) is robust as it occurs among different marine reserve sizes with hypothetical parameter values (Figure 1a‐f) and with parameters estimated from the West Coast groundfish fisheries (Figure 1g‐n).

With the random initial density, the initial fluctuations and the inconsistency between the population densities and fisheries yields increase the difficulty of precise prediction in fisheries management. In comparison with the dynamics that initial densities both inside and outside the reserves are very low, the random initial density increases the fluctuations in the population density both inside and outside the marine reserve (Figure 2a, c, e, g, i, k, and m, i.e., the left panels in Figure 2) and the fluctuations in the fisheries yields (Figure 2b, d, f, h, j, l, and n, i.e., the right panels in Figure 2). Moreover, the transient dynamics are not consistent between the population densities and fisheries yields (The left panels vs. the right panels in Figure 2). Nevertheless, the fluctuations disappear when the dynamics approach equilibrium stable states, which suggests that precise predictions in fisheries may be possible at that moment.

Sensitivity analysis shows that how close (*θ*) and how fast (*ρ*) initial state converges to equilibrium depend on the variation in the marine reserve size and the escapement rate (strongly related to fishing effort) for the strong stocks (Figure 3). In general, the marine reserve size and the escapement rate have a reverse effect on transient metrics *θ* and *ρ* by controlling the variations in all other parameters (Figure 3). The initial conditions far away from a stable equilibrium state (i.e., high value of *θ*) can increase the amplitude of oscillations (shown at the beginning of the trajectories of the transient population dynamics in Figure 2) and thus increase the variance in population trajectories. A low convergence rate (i.e., low value of *ρ*) to an asymptotic equilibrium state means the convergence of population density and fisheries yields to stable level is slow which will increase the transient duration and thus decrease the detectability in fisheries management. Therefore, predicting trajectories of population density and fisheries yields will be difficult if the marine reserve size (*c*) is small and the escapement rate (*E*) is high because of high *θ* and low *ρ* (Figure 3). The reverse effect of marine reserve size (*c*) and escapement rate (*E*) on *θ* (or *ρ*) is derived from the fact that the escapement rate is a monotonously decreasing function of marine reserve size under the weak stock persistence condition (see the analytical derivation in the section of “Demonstration that *E* is decreasing in *c*” in Appendix S1).

## 7 DISCUSSION

Permanent marine reserves are an effective way to maintain fisheries yields without sacrificing the persistence of endangered species and thus provide new methods for solving an important global issue in fisheries management (i.e., fisheries bycatch) (Hastings et al., 2017). However, one of the challenges still faced in fisheries bycatch assessments is the lack of theoretical predictions in the transient phenomena, especially when the population dynamics of the target species are strongly correlated with the timescales. The transient analyses in this paper show that the transient emergence of fisheries yields of target species does not depend on the transient emergence of the population density both inside and outside the marine reserve (Figure 2). Thus, the occurrence of transient phenomena in one variable cannot be used to predict the other for the limited case that bycatch species are persistent.

One of the most important procedures in fisheries management is collecting information by monitoring the population dynamics of the target species, and the monitoring information is then used to achieve the goal of maximizing fisheries yields and sustainably maintaining the species. However, this intuitive management method (monitoring first and then setting fishing policies based on the monitoring information) is based on an easily ignored assumption that there is transient consistency between population dynamics and fisheries yields. Therefore, it makes sense to use one variable to predict the other. However, the analysis results in this paper suggest that fisheries yields are unpredictable even with sufficient monitoring information about the population dynamics because fisheries yields can exhibit great variations and are thus unstable even if the population density dynamics are deterministic and only slightly vary (i.e., the transient inconsistency between them) (Figure 2), which is consistent with the emerging paradigm monitoring reserve effects are highly sensitive to the variables that measured (Moffitt et al., 2013). Thus, the transient dynamics of the population density of the strong stock cannot be used to predict the transient dynamics of the fisheries yields of the strong stock under conditions in which the weak stock is persistent. The transient dynamics of fisheries yields should be investigated even if there is no transient phenomenon in the population density dynamics because of the transient inconsistency between fisheries yields and population density. The transient inconsistency may be caused by the parameter of adult survival (*a*) in Equations 1 and 2. If the survivorship of adults is zero, population density inside and outside the marine reserve would have linear relationships (substitute Equation 1 into Equation 2), which indicates that the relative variation between population density inside and outside the marine reserve in each iteration is fixed and they will simultaneously increase or decrease in each iteration. However, the ratio between population density inside and outside the marine reserve varies in each iteration if the survivorship of adults does not equal zero, which leads to a complex relationship between them and would be unpredictable after iteration happens. In addition, fisheries yields are calculated by the interplay between population density inside and outside the marine reserve. Thus, the transient inconsistency between fisheries yields and population densities occurs.

Fisheries yields of the strong stock under persistence of the weak stock have been investigated to show great advantages of marine reserves (Hastings et al., 2017), which provide theoretical predictions in fisheries management. However, the fact that predicting the strong stock yields based on population density is probably impossible if the population density is far away from the stable asymptotic equilibrium as the transient analysis here suggests that the detectability is decreased by the transient inconsistency between population density and fisheries yields. Thus, the transient analyses here extend the previous work (Hastings et al., 2017) to provide cautions in fisheries management by emphasizing the conditions (such as approach stable equilibrium) under which population density can be used to predict fisheries yields.

The analysis results show that densities inside reserves increase with the decreasing of marine reserve size (Figures 1 and 2), which can be explained by the weak stock persistence condition. The same escapement rate (*E*) and marine reserve size (*c*) in the theoretical framework for both strong and weak stock species suggest that the strong stock species must be persistent if weak stock species can persist. Therefore, the weak stock persistence condition indeed can also make sure the persistence of the strong stock species (in fact, the strong stock can persist even under some more stringent cases when the weak stock cannot persist). The persistence condition in the model studied here leads to high densities inside the reserves when marine reserve size is small. In addition, the negative relationship between densities inside reserves and marine reserve size is true for all but situation 4 (Figures 1 and 2). This derives from the fact that, with lowest survival in situation 4, the weak stock species is bocaccio which in fact is not a true weak stock as predicted by previous studies (Hastings et al., 2017).

Although the transient inconsistency between fisheries yields and population densities hampers the precise prediction in fisheries management, the results show it is possible to make a correct decision when population dynamics approach the equilibrium stable states. This implication provides a method to achieve precise predictions in fisheries management: reducing the uncertainty in prediction through shortening the transient duration so that the dynamics can approach the equilibrium stable states as soon as possible. The sensitivity analyses indicate that a short transient duration may occur through increasing the marine reserve size and decreasing the escapement rate (Figure 3). The implications suggest that the combined management of establishing small marine reserves and traditional fishery controls by reducing fishing effort (or conversely, establishing large marine reserve when the fishery is heavily exploited) has many advantages in fisheries management including reducing the uncertainty in fisheries prediction and maintain sustainably harvested yields. My analysis results are consistent with some previous studies which suggest that spatial closures in marine system associated with fishing effort control can yield conservation and fishery benefits in the long term (Hopf et al., 2016b). Moreover, previous relevant studies show that either large marine reserves or weak fishing effort can reduce the time period of yield recovery after the fishery was heavily exploited (Hopf et al., 2016a), which suggests a short transient duration and are consistent with the analysis here. However, the studies of the two‐species system here simultaneously suggest that weak fishing effort should correspond to small marine reserves, while large marine reserves correspond to strong fishing effort if the combined management of reserves and fishing control is used to achieve both conservation and fisheries goals.

Investigating how the life history of the target fished species regulates transient population dynamics is important in predicting detectable timeline for adaptive management of marine protected areas (Kaplan et al., 2019; Nickols et al., 2019; White et al., 2013). Previous work suggests that fish individuals with higher survival (White et al., 2013), lower recruitment years before marine protected area establishment (Nickols et al., 2019), and increasing recruitment variability (Kaplan et al., 2019) can lead to longer transient dynamics. However, the survival and recruitment of the target fished species might have little effect on the oscillations of the transient dynamics of the strong–weak stock two‐species system as suggested by the analyses with different survivals (Figure S1) and different recruitments (Figure S2) of the target strong species. The inconsistent conclusions derive from the fact that the weak–strong stock two‐species system studied here does not include age structure which is suggested to be an important factor that causing transients (White et al., 2013). Instead, the transients observed here are caused by saddle points where densities inside and outside marine reserves are very low.

The theoretical framework that is used for transient analysis in this study does not take time delays (i.e., time lags, e.g., the lags of reserve effects on fisheries yields) into account. Although a time delay increases the difficulty of studying a specific system, it can also cause transient phenomena even for some low‐dimensional systems such as a simple two‐species model (Hastings et al., 2018; Morozov et al., 2019). Time delays should be taken into consideration, especially for some very common cases in fishery. For example, the effect of fishing activities on the population growth rate may not be exhibited immediately after the implementation of harvesting. Similarly, increase in reproductive output of populations may not happen immediately after the implementation of marine reserve, and trajectories of transient responses of population density may be flat or even decreasing during the short‐term predictions, which is different from long‐term predictions (Nickols et al., 2019). Another issue that deserves further attention is the transient dynamics of the weak stock. In this research, only the transient dynamics of the strong stock are considered based on the assumption that the weak stock is persistent. However, simple predications in the transients of the weak stock can be achieved based on the transients of the strong stock. This derives from two points: (a) The dynamic models are in fact the same for both strong and weak stock species although the parameter values with biological meaning are different; and (b) the analysis is based on the implicit assumption that there is no interplay (i.e., ecological connection) between the density of the strong stock and the density of the weak stock. This suggests that the transient dynamics of the bycatch species will be much similar to that of the strong stock species studied here. Future directions are developing new theoretical frameworks by considering the interplay of population density between strong and weak stock species so that to deepen understandings of how transients of the weak stock affect the transients of the strong stock or vice versa. In addition, the weak stock condition in Equation 5 may be not sufficient in real‐world systems although it is necessary. This is derived from the fact that the weak stock condition in Equation 5 cannot exclude the situation that the population density of the weak stock is very low both inside and outside the marine reserve. If the weak stock stays at very low levels, it easily goes extinct because of the stochastic environmental factors and the Allee effects in natural systems.

## CONFLICT OF INTEREST

There are no competing interests.

## AUTHOR CONTRIBUTION

**Renfei Chen:** Conceptualization (lead); Formal analysis (lead); Funding acquisition (lead); Methodology (lead); Project administration (lead); Software (lead); Writing‐original draft (lead); Writing‐review & editing (lead).

## ETHICAL APPROVAL

This article has no ethic problems.

## Supporting information

AppendixS1

FigS1

FigS2

## Data Availability

This article has no additional data, and there is no need to deposit data to a public repository.
