# Supplementary material for: Transient inconsistency between population density and fisheries yields without bycatch species extinction
Source: Ecol Evol. 2020 Oct 10;10(21):12372–84. doi: 10.1002/ece3.6868 (PMC7663084; doi:10.1002/ece3.6868)
Supplement: Supplementary file 1 — AppendixS1 [file ECE3-10-12372-s001.docx]

**Title: Transient inconsistency between population density and fisheries yields under bycatch persistence**

Renfei Chen^1^*

^1^ School of Life Science, Shanxi Normal University, Yaodu, Linfen, 041000, China.

*Corresponding author: Renfei Chen, [chenrf@sxnu.edu.cn](mailto:chenrf@sxnu.edu.cn)

ORCiD IDs: [https://orcid.org/0000-0003-2541-3365](https://mc.manuscriptcentral.com/oikos)

**Analytical equilibrium solution for the strong stock**

For the density dynamics described by Eqs. 1 and 2 in the main text, the equilibrium states occur when $n_{t+1}^{R}=n_{t}^{R}$, and the following equations can be achieved with *f*() representing the Beverton–Holt functional form ($f\left( n \right)=\frac{\alpha n}{1+\frac{n}{\beta}}=\frac{\alpha\beta n}{\beta+n}$):

$$m\left( cn_{t}^{R}+\left( 1-c \right)n_{t}^{O} \right)\alpha\beta=n_{t}^{R}\left( 1-a \right)\left[ \beta+m\left( cn_{t}^{R}+\left( 1-c \right)n_{t}^{O} \right) \right] (A.1)$$

$$m\left( cn_{t}^{R}+\left( 1-c \right)n_{t}^{O} \right)\alpha\beta E=n_{t}^{o}\left( 1-Ea \right)\left[ \beta+m\left( cn_{t}^{R}+\left( 1-c \right)n_{t}^{O} \right) \right] (A.2)$$

Assuming $m\left( cn_{t}^{R}+\left( 1-c \right)n_{t}^{O} \right)\neq0$ (If $m\left( cn_{t}^{R}+\left( 1-c \right)n_{t}^{O} \right)=0$, $n_{t}^{R}=n_{t}^{O}=0$), the quotient of Eq. A.1 and Eq. A.2 is:

$$\frac{1}{E}=\frac{n_{t}^{R}}{n_{t}^{O}}\frac{1-a}{\left( 1-Ea \right)} (A.3)$$

i.e.,

$$n_{t}^{O}=n_{t}^{R}\frac{(1-a)E}{\left( 1-Ea \right)} (A.4)$$

Substitute Eq. A.4 into Eq. A.1:

$$\left( c+\left( 1-c \right)\frac{(1-a)E}{\left( 1-Ea \right)} \right)m\alpha\beta n_{t}^{R}=n_{t}^{R}\left( 1-a \right)\left[ \beta+mn_{t}^{R}\left( c+\left( 1-c \right)\frac{(1-a)E}{\left( 1-Ea \right)} \right) \right] (A.5)$$

Therefore, $n_{t}^{R}$ has two solutions: $n_{t}^{R}=0$ and $n_{t}^{R}=\frac{\alpha\beta}{1-a}-\frac{\beta\left( 1-Ea \right)}{mc\left( 1-Ea \right)+m\left( 1-c \right)\left( 1-a \right)E}$.

According to Eq. A.4, $n_{t}^{O}=0$ and $n_{t}^{O}=\frac{\alpha\beta E}{1-Ea}-\frac{\beta E\left( 1-a \right)}{mc+m\left( 1-a-c \right)E}$. For a special case of the system (Eq. 1 and Eq. 2), if $n_{t}^{R}=0$, then $n_{t}^{O}=0$. If $n_{t}^{O}=0$, then $n_{t}^{R}=(mc\alpha\beta+(a-1)\beta)/(1-a)mc$ under the condition that $E=0,a\neq1,c\neq0$, or $n_{t}^{R}=0$. If $a=1$, then $n_{t}^{R}=n_{t}^{O}=0$. Accordingly, there are three scenarios for the strong stock density at equilibria:

**Scenario 1**

$n_{t}^{R}=0$ $(A.6)$

$$n_{t}^{O}=0 (A.7)$$

**Scenario 2**

$$n_{t}^{R}=\frac{mc\alpha\beta+\left( a-1 \right)\beta}{\left( 1-a \right)mc} (A.8)$$

$$n_{t}^{O}=0 (E=0,a\neq1,c\neq0)\left( A.9 \right)$$

**Scenario 3**

$$n_{t}^{R}=\frac{\alpha\beta}{1-a}-\frac{\beta\left( 1-Ea \right)}{mc\left( 1-Ea \right)+m\left( 1-c \right)\left( 1-a \right)E} (A.10)$$

$$n_{t}^{O}=\frac{\alpha\beta E}{1-Ea}-\frac{\beta E\left( 1-a \right)}{mc+m\left( 1-a-c \right)E} (a\neq1)\left( A.11 \right)$$

**The value ranges for the parameters**

The density inside and outside marine reserves should not be negative (i.e., $n_{t}^{R}\geq0,n_{t}^{O}\geq0$). Therefore, we have the following inequation for scenario 2:

$$\frac{mc\alpha\beta+\left( a-1 \right)\beta}{\left( 1-a \right)mc}\geq0 (A.12)$$

The condition for scenario 3 is as follows:

$$\frac{\alpha\beta}{1-a}-\frac{\beta\left( 1-Ea \right)}{mc\left( 1-Ea \right)+m\left( 1-c \right)\left( 1-a \right)E} \geq0(A.13)$$

$$\frac{\alpha\beta E}{1-Ea}-\frac{\beta E\left( 1-a \right)}{mc+m\left( 1-a-c \right)E} \geq0(A.14)$$

When the escapement rate *E* is 1, there are no fishing activities, and all the analyses have no biological meaning. Thus, $0\leq E<1$. According to the weak stock persistence condition (i.e., Eq. 5), we have:

$$0\leq\frac{a_{w}-1+\alpha_{w}m_{w}c}{\left( a_{w}-1 \right)\left( a_{w}+\alpha_{w}m_{w} \right)+\alpha_{w}m_{w}c}<1 (A.15)$$

When Eq. A.12 is combined with Eq. A.15, the condition for scenario 2 is:

$$\frac{1-a}{m\alpha}\leq c\leq\frac{1-a_{w}}{\alpha_{w}m_{w}} (A.16)$$

The condition for scenario 3 is as follows:

$$\frac{E\left( a-1 \right)}{1-E}+\frac{(1-a)(1-Ea)}{m\alpha(1-E)}\leq c\leq\frac{1-a_{w}}{\alpha_{w}m_{w}} (A.17)$$

**Transient analysis by finding a saddle point**

The Jacobian matrix of Eq. 1 and Eq. 2 is:

$$J=\left[ \begin{matrix} a-1+\frac{mc\alpha\beta^{2}}{\left[ \beta+\mathrm{mc}n_{t}^{R}+m\left( 1-c \right)n_{t}^{O} \right]^{2}} & \frac{m\left( 1-c \right)\alpha\beta^{2}}{\left[ \beta+\mathrm{mc}n_{t}^{R}+m\left( 1-c \right)n_{t}^{O} \right]^{2}} \\ \frac{Emc\alpha\beta^{2}}{\left[ \beta+\mathrm{mc}n_{t}^{R}+m\left( 1-c \right)n_{t}^{O} \right]^{2}} & Ea-1+\frac{Em\left( 1-c \right)\alpha\beta^{2}}{\left[ \beta+\mathrm{mc}n_{t}^{R}+m\left( 1-c \right)n_{t}^{O} \right]^{2}} \end{matrix} \right] (A.18)$$

For scenario 1, $n_{t}^{R}=n_{t}^{O}=0$. Thus, the Jacobian matrix turns out to be:

$$J=\left[ \begin{matrix} a-1+mc\alpha& m\left( 1-c \right)\alpha\\ Emc\alpha& Ea-1+Em\left( 1-c \right)\alpha\end{matrix} \right] (A.19)$$

If there is a saddle point for the system, then one of the eigenvalues of the Jacobian matrix is positive and the other is negative. Thus:

$$\lambda_{1}\lambda_{2}=\left( 1-a \right)\left( 1-Ea \right)+m\alpha\left( Ea-E+Ec-c \right)<0 (A.20)$$

where $\lambda_{1}$ and $\lambda_{2}$ are eigenvalues of the Jacobian matrix (Eq. A.18). Therefore, for scenario 1, the condition that meets a saddle point is:

$$\frac{m\alpha}{1-a}>\frac{1-Ea}{c+E(1-a-c)} (A.21)$$

For scenario 2, Eq. A.8 and Eq. A.9 are substituted into Eq. A.18, and the condition that meets a saddle point is:

$$\lambda_{1}\lambda_{2}=\left( 1-a \right)\left( 1-Ea \right)-\frac{E\left( 1-c \right)\left( 1-a \right)^{3}}{mc^{2}\alpha}-\frac{\left( 1-Ea \right)\left( 1-a \right)^{2}}{mc\alpha}<0 (A.22)$$

Note that the escapement rate (i.e. *E*) is zero in scenario 2 (please see Eq. A.9). Thus, Eq. A.22 will be:

$$\lambda_{1}\lambda_{2}=\left( 1-a \right)-\frac{\left( 1-a \right)^{2}}{mc\alpha}<0 (A.23)$$

Meanwhile, $a\neq1$in Eq. A.9. Thus, by removing $1-a$ in Eq. A.23, we have $c<\frac{1-a}{m\alpha} (A.24)$

For scenario 3, Eq. A.10 and Eq. A.11 are substituted into Eq. A.18, and the Jacobian matrix turns out to be:

$$J=\left[ \begin{matrix} a-1+\frac{c}{\alpha m}{[\frac{\left( 1-Ea \right)\left( 1-a \right)}{c+(1-c-a)E}]}^{2} & {\frac{\left( 1-c \right)}{\alpha m}[\frac{\left( 1-Ea \right)\left( 1-a \right)}{c+(1-c-a)E}]}^{2} \\ \frac{Ec}{\alpha m}{[\frac{\left( 1-Ea \right)\left( 1-a \right)}{c+(1-c-a)E}]}^{2} & Ea-1+{\frac{E\left( 1-c \right)}{\alpha m}[\frac{\left( 1-Ea \right)\left( 1-a \right)}{c+(1-c-a)E}]}^{2} \end{matrix} \right]\left( A.25 \right)$$

The condition that meets a saddle point is:

$$\lambda_{1}\lambda_{2}=\left( 1-a \right)\left( 1-Ea \right)+\frac{\left( a-1 \right)E\left( 1-c \right)+c\left( Ea-1 \right)}{\alpha m}\left[ \frac{\left( 1-Ea \right)\left( 1-a \right)}{c+\left( 1-c-a \right)E} \right]^{2}$$

$$=\left( 1-a \right)\left( 1-Ea \right)-\frac{\left[ \left( 1-Ea \right)\left( 1-a \right) \right]^{2}}{\alpha m[c+\left( 1-c-a \right)E]}<0 (A.26)$$

If $\left( 1-a \right)\left( 1-Ea \right)=0$, then $\lambda_{1}\lambda_{2}=0$, and the inequation A.26 cannot hold. Thus, by removing $\left( 1-a \right)\left( 1-Ea \right)$ in the inequation A.26, we can obtain:

$$\frac{\left( 1-Ea \right)\left( 1-a \right)}{\alpha m[c+\left( 1-c-a \right)E]}>1 (A.27)$$

Thus,

$$\frac{m\alpha}{1-a}<\frac{1-Ea}{c+E(1-a-c)} (A.28)$$

Clearly, Eq. A.16 and Eq. A.24 as well as Eq. A.17 and Eq. A.28 cannot hold at the same time, which means that there is no saddle point for scenarios 2 and 3. Accordingly, the saddle point of the system occurs only at (0,0) under appropriate conditions.

**Transient analysis by considering fast-slow systems**

If the strong stock species has a much higher growth rate than the weak stock species, then the system consist of the weak and strong stock species can be analyzed as a fast-slow system. Accordingly, we analyze the fast components of the system consist of the weak and strong stock species with a widely accepted method (Rinaldi and Scheffer 2000) to predict whether there are Hopf bifurcations in the population dynamics of the strong stock. The equivalent condition of Hopf bifurcations is that the trace of the Jacobian matrix (Eq. A.18) is zero, and the determinant of this matrix is positive. That is:

$$trace\left[ J \right]=0,\det\left[ J \right]>0 (A.29)$$

Similarly, this part is discussed with three scenarios according to the density inside and outside marine reserves at equilibria. For scenario 1, Eq. A.29 is identical to

$$a-1+mc\alpha+ Ea-1+Em\left( 1-c \right)\alpha=0 (A.30)$$

$$(a-1)(Ea-1)+(a-1)Em\left( 1-c \right)\alpha+mc\alpha(Ea-1)>0 (A.31)$$

Substitute Eq. A.30 into the inequation A.31,

$$(a-1)(Ea-1)+(1-a)(a-1+mc\alpha+ Ea-1)+mc\alpha(Ea-1)$$

$$=\left( a-1 \right)\left( Ea-1 \right)+\left( 1-a \right)\left( a-1+mc\alpha+Ea-1 \right)+mc\alpha\left( Ea-1 \right)$$

$$=\left( 1-a \right)\left( a-1+mc\alpha\right)+mc\alpha\left( Ea-1 \right)$$

$$={-\left( 1-a \right)}^{2}+mc\alpha\left( Ea-a \right)$$

$$={-\left( 1-a \right)}^{2}+mc\alpha a\left( E-1 \right)>0$$

For scenario 2, Eq. A.29 is identical to

$$\left( a-1 \right)+\frac{\left( 1-a \right)^{2}}{mc\alpha}+\left( Ea-1 \right)+\frac{E\left( 1-c \right)\left( 1-a \right)^{2}}{mc^{2}\alpha}=0 (A.32)$$

$$\left( a-1 \right)\left( Ea-1 \right)-\frac{E\left( 1-c \right)\left( 1-a \right)^{3}}{mc^{2}\alpha}-\frac{\left( 1-Ea \right)\left( 1-a \right)^{2}}{mc\alpha}>0 (A.33)$$

Remove$1-a$ ($a\neq1$ in scenario 2) in the inequation A.33 and substitute Eq. A.32 into inequation A.33 to achieve:

$$\left( 1-Ea \right)-\frac{E\left( 1-c \right)\left( 1-a \right)^{2}}{mc^{2}\alpha}-\frac{\left( 1-Ea \right)(1-a)}{mc\alpha}$$

$$=\left( a-1 \right)+\frac{\left( 1-a \right)^{2}}{mc\alpha}-\frac{\left( 1-Ea \right)(1-a)}{mc\alpha}$$

$$=\left( a-1 \right)-\frac{\left( a-Ea \right)(1-a)}{mc\alpha}$$

$$=(1-a)[-1-\frac{a\left( 1-E \right)}{mc\alpha}]>0$$

For scenario 3, Eq. A.29 is identical to

$$a+Ea-2+{\frac{c+E\left( 1-c \right)}{\alpha m}[\frac{\left( 1-Ea \right)\left( 1-a \right)}{c+(1-c-a)E}]}^{2}=0 (A.34)$$

$$(a-1)(Ea-1)+{\frac{c(Ea-1)+E\left( 1-c \right)(a-1)}{\alpha m}[\frac{\left( 1-Ea \right)\left( 1-a \right)}{c+(1-c-a)E}]}^{2}>0 (A.35)$$

Substitute Eq. A.34 into the inequation A.35,

$$\left( a-1 \right)\left( Ea-1 \right)+\frac{2-a-Ea}{c+E\left( 1-c \right)}\left[ c\left( Ea-1 \right)+E\left( 1-c \right)\left( a-1 \right) \right]$$

$$=\left( a-1 \right)\left( Ea-1 \right)+\frac{2-a-Ea}{c+E\left( 1-c \right)}\left[ -c+Ea-E+Ec \right]$$

$$=\left( a-1 \right)\left( Ea-1 \right)+\frac{2-a-Ea}{c+E\left( 1-c \right)}\left[ Ea-c-E+Ec \right]$$

$$=\left( a-1 \right)\left( Ea-1 \right)+\frac{2-a-Ea}{c+E\left( 1-c \right)}\left[ Ea-(c+E\left( 1-c \right)) \right]$$

$$=\left( a-1 \right)\left( Ea-1 \right)+\frac{(2-a-Ea)Ea}{c+E\left( 1-c \right)}-(2-a-Ea)$$

$$=Ea^{2}-1+\frac{(2-a-Ea)Ea}{c+E\left( 1-c \right)}$$

$$=\frac{(Ea^{2}-1-E^{2}a^{2}+E)c-E(1-2a+a^{2})}{c+E\left( 1-c \right)}$$

$$=\frac{(Ea^{2}-1)(1-E)c-E{(1-a)}^{2}}{c+E\left( 1-c \right)}>0$$

Thus, for scenarios 1, 2 and 3, the calculation results based on Eq. A.29 for the condition of Hopf bifurcations are exhibited as the following three inequations, respectively:

$$\left( a-1 \right)^{2}+mc\alpha a\left( 1-E \right)<0 (A.36)$$

$$1<-\frac{a\left( 1-E \right)}{mc\alpha} (A.37)$$

$$\left( 1-E \right)c\left( Ea^{2}-1 \right)>E\left( 1-a \right)^{2} (A.38)$$

all of which are impossible (for example, in scenario 3, the left part of Eq. A.38 is negative while the right part is positive, which is impossible). Accordingly, when the theoretical framework is regarded as a fast-slow system, there is no Hopf bifurcation in the strong stock population dynamics.

**Demonstration that *E* is decreasing in *c***

Based on Eq. 5 in the main text, we can calculate the derivation of *E* in *c* to obtain,

$$E^{'}(c)=\frac{\alpha_{w}m_{w}[\left( a_{w}-1 \right)\left( a_{w}+\alpha_{w}m_{w} \right)+\alpha_{w}m_{w}c]-[a_{w}-1+\alpha_{w}m_{w}c]\alpha_{w}m_{w}}{{[\left( a_{w}-1 \right)\left( a_{w}+\alpha_{w}m_{w} \right)+\alpha_{w}m_{w}c]}^{2}}$$

$$=\frac{\alpha_{w}m_{w}\left( a_{w}-1 \right)\left( a_{w}+\alpha_{w}m_{w}-1 \right)}{{[\left( a_{w}-1 \right)\left( a_{w}+\alpha_{w}m_{w} \right)+\alpha_{w}m_{w}c]}^{2}}$$

Previous researches indicate that $a_{w}+\alpha_{w}m_{w}-1>0$ (please see relevant information in the inequation 17 in Hastings et al. 2017). In addition, $a_{w}-1<0$. Thus, $E^{'}(c)<0$, which indicates that *E* is a decreasing function of *c*.

**Reference**

Hastings, A., S. D. Gaines, and C. Costello. 2017. Marine reserves solve an important bycatch problem in fisheries. Proceedings of the National Academy of Sciences USA **114**:8927-8934.

Rinaldi, S. and M. Scheffer. 2000. Geometric analysis of ecological models with slow and fast processes. Ecosystems **3**:507-521.
