# Supplementary material for: Transient inconsistency between population density and fisheries yields without bycatch species extinction
Source: Ecol Evol. 2020 Oct 10;10(21):12372–84. doi: 10.1002/ece3.6868 (PMC7663084; doi:10.1002/ece3.6868)
Supplement: Supplementary file 2 — FigS1 [file ECE3-10-12372-s002.pdf]

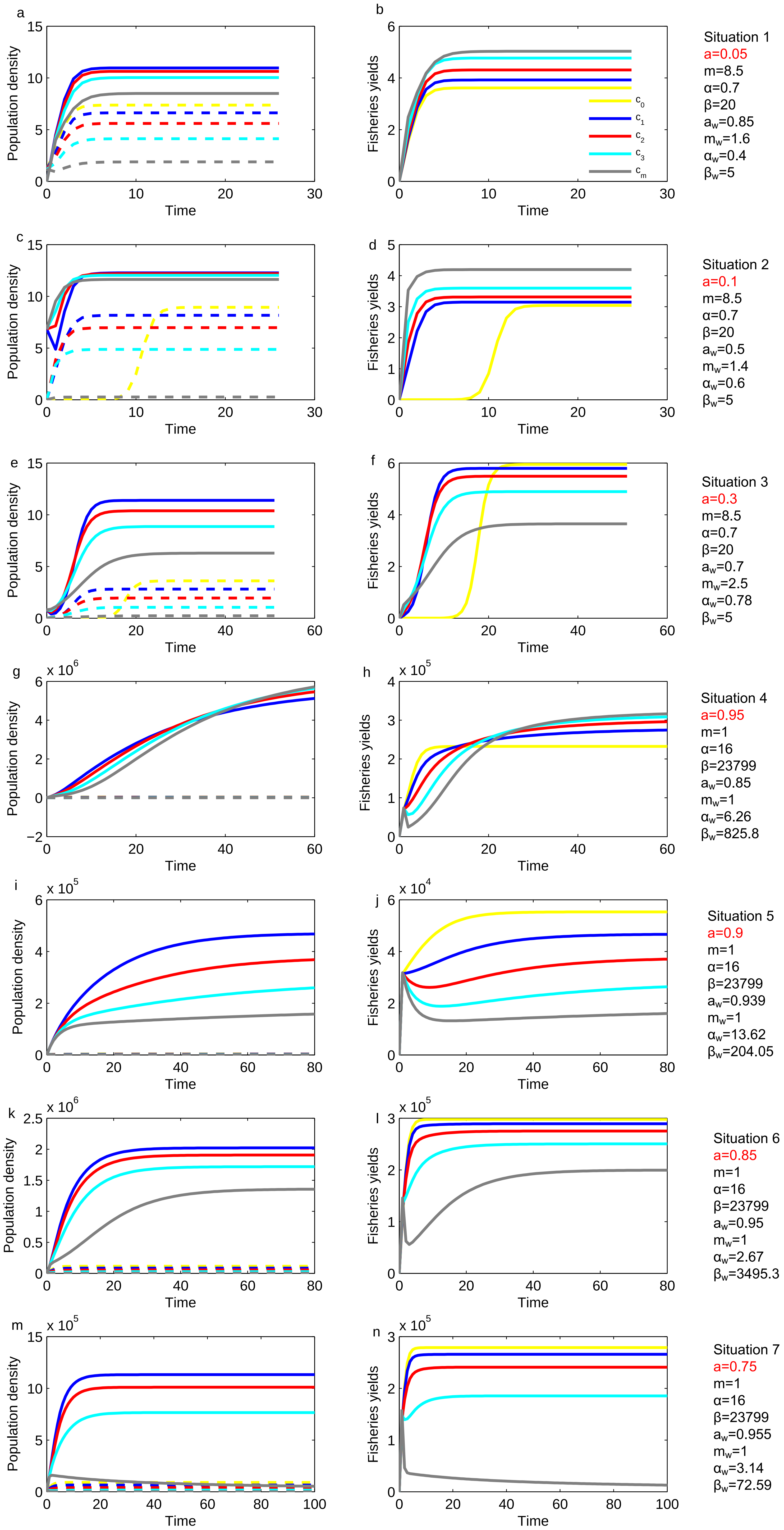

a
Population density
Time
b
Fisheries yields
Time
c_0
c_1
c_2
c_3
c_m
Situation 1
a=0.05
m=8.5
α=0.7
β=20
a_w=0.85
m_w=1.6
$α_w$=0.4
$β_w$=5
c
Population density
Time
d
Fisheries yields
Time
Situation 2
a=0.1
m=8.5
α=0.7
β=20
a_w=0.5
m_w=1.4
$α_w$=0.6
$β_w$=5
e
Population density
Time
f
Fisheries yields
Time
Situation 3
a=0.3
m=8.5
α=0.7
β=20
a_w=0.7
m_w=2.5
$α_w$=0.78
$β_w$=5
g
x 10^6
Population density
Time
h
x 10^5
Fisheries yields
Time
Situation 4
a=0.95
m=1
α=16
β=23799
a_w=0.85
m_w=1
$α_w$=6.26
$β_w$=825.8
i
x 10^5
Population density
Time
j
x 10^4
Fisheries yields
Time
Situation 5
a=0.9
m=1
α=16
β=23799
a_w=0.939
m_w=1
$α_w$=13.62
$β_w$=204.05
k
x 10^6
Population density
Time
l
x 10^5
Fisheries yields
Time
Situation 6
a=0.85
m=1
α=16
β=23799
a_w=0.95
m_w=1
$α_w$=2.67
$β_w$=3495.3
m
x 10^5
Population density
Time
n
x 10^5
Fisheries yields
Time
Situation 7
a=0.75
m=1
α=16
β=23799
a_w=0.955
m_w=1
$α_w$=3.14
$β_w$=72.59
