# Supplementary material for: Transient inconsistency between population density and fisheries yields without bycatch species extinction
Source: Ecol Evol. 2020 Oct 10;10(21):12372–84. doi: 10.1002/ece3.6868 (PMC7663084; doi:10.1002/ece3.6868)
Supplement: Supplementary file 3 — FigS2 [file ECE3-10-12372-s003.pdf]

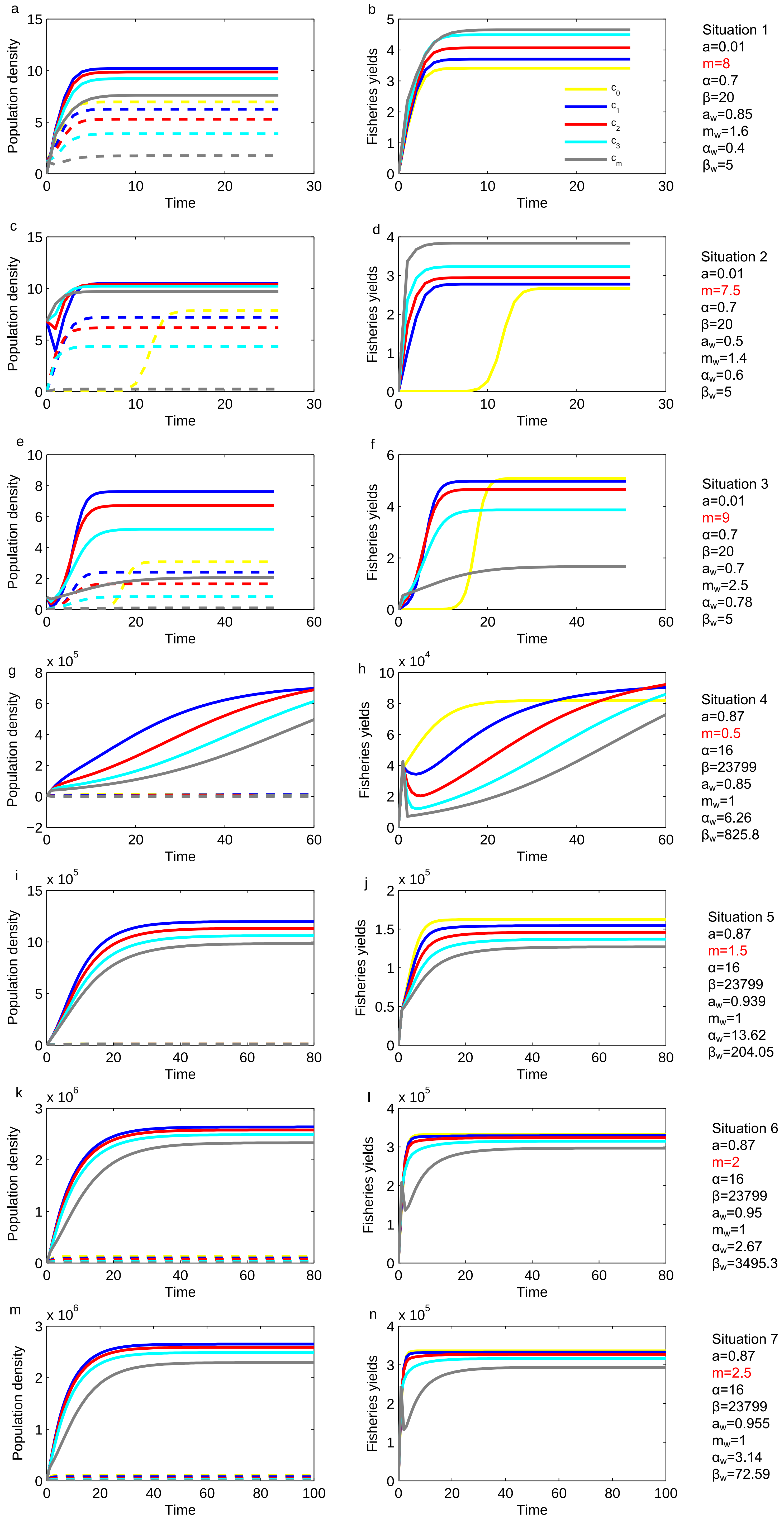

a
Population density
Time
b
Fisheries yields
Time
c0
c1
c2
c3
cm
Situation 1
a=0.01
m=8
α=0.7
β=20
aw=0.85
mw=1.6
αw=0.4
βw=5
c
Population density
Time
d
Fisheries yields
Time
Situation 2
a=0.01
m=7.5
α=0.7
β=20
aw=0.5
mw=1.4
αw=0.6
βw=5
e
Population density
Time
f
Fisheries yields
Time
Situation 3
a=0.01
m=9
α=0.7
β=20
aw=0.7
mw=2.5
αw=0.78
βw=5
g
x 10^5
Population density
Time
h
x 10^4
Fisheries yields
Time
Situation 4
a=0.87
m=0.5
α=16
β=23799
aw=0.85
mw=1
αw=6.26
βw=825.8
i
x 10^5
Population density
Time
j
x 10^5
Fisheries yields
Time
Situation 5
a=0.87
m=1.5
α=16
β=23799
aw=0.939
mw=1
αw=13.62
βw=204.05
k
x 10^6
Population density
Time
l
x 10^5
Fisheries yields
Time
Situation 6
a=0.87
m=2
α=16
β=23799
aw=0.95
mw=1
αw=2.67
βw=3495.3
m
x 10^6
Population density
Time
n
x 10^5
Fisheries yields
Time
Situation 7
a=0.87
m=2.5
α=16
β=23799
aw=0.955
mw=1
αw=3.14
βw=72.59
